# Accuracy and precision of end-expiratory lung-volume measurements by automated nitrogen washout/washin technique in patients with acute respiratory distress syndrome

**DOI:** 10.1186/cc10587

**Published:** 2011-12-07

**Authors:** Jean Dellamonica, Nicolas Lerolle, Cyril Sargentini, Gaetan Beduneau, Fabiano Di Marco, Alain Mercat, Jean-Christophe M Richard, Jean-Luc Diehl, Jordi Mancebo, Jean-Jacques Rouby, Qin Lu, Gilles Bernardin, Laurent Brochard

**Affiliations:** 1 Réanimation Médicale, AP-HP, Centre Hospitalier Albert Chenevier, Henri Mondor, avenue Marechal de Lattre de Tassigny, Créteil, 94000, France; 2 Réanimation Médicale, CHU de Nice, Hôpital L'Archet, Université de Nice Sophia Antipolis, Route de St Antoine de Ginestière, Nice 06200, France; 3 Réanimation Médicale, AP-HP, Hôpital Européen Georges Pompidou, rue Leblanc, Paris 75015, France; 4 Réanimation Médicale, CHU Angers, rue Larrey, Angers 49100, France; 5 Réanimation Médicale & UPRES EA 3830, CHU Charles Nicolle, rue Germont, Rouen 76031, France; 6 Pneumologia Ospedale San Paolo, Universita degli Studi di Milano, via A. di Rudini 8, Milano 20142, Italy; 7 Servei de Medicina Intensiva, Hospital de Sant Pau, C. Sant Quinti 89, Barcelona, 08041, Spain; 8 Réanimation Polyvalente, AP-HP, Hôpital Pitié Salpêtrière, UPMC, Université Paris 6, Boulevard de l'Hôpital, Paris 75014, France; 9 INSERM U-955, Université Paris EST, avenue Marechal de Lattre de Tassigny, Créteil 94000, France; 10 Intensive Care Department, University Hospital and University of Geneva, rue Gabrielle Perret-Gentil, Geneva 1211, Switzerland

## Abstract

**Introduction:**

End-expiratory lung volume (EELV) is decreased in acute respiratory distress syndrome (ARDS), and bedside EELV measurement may help to set positive end-expiratory pressure (PEEP). Nitrogen washout/washin for EELV measurement is available at the bedside, but assessments of accuracy and precision in real-life conditions are scant. Our purpose was to (a) assess EELV measurement precision in ARDS patients at two PEEP levels (three pairs of measurements), and (b) compare the changes (Δ) induced by PEEP for total EELV with the PEEP-induced changes in lung volume above functional residual capacity measured with passive spirometry (ΔPEEP-volume). The minimal predicted increase in lung volume was calculated from compliance at low PEEP and ΔPEEP to ensure the validity of lung-volume changes.

**Methods:**

Thirty-four patients with ARDS were prospectively included in five university-hospital intensive care units. ΔEELV and ΔPEEP volumes were compared between 6 and 15 cm H_2_O of PEEP.

**Results:**

After exclusion of three patients, variability of the nitrogen technique was less than 4%, and the largest difference between measurements was 81 ± 64 ml. ΔEELV and ΔPEEP-volume were only weakly correlated (*r^2 ^*= 0.47); 95% confidence interval limits, -414 to 608 ml). In four patients with the highest PEEP (≥ 16 cm H_2_O), ΔEELV was lower than the minimal predicted increase in lung volume, suggesting flawed measurements, possibly due to leaks. Excluding those from the analysis markedly strengthened the correlation between ΔEELV and ΔPEEP volume (*r^2 ^*= 0.80).

**Conclusions:**

In most patients, the EELV technique has good reproducibility and accuracy, even at high PEEP. At high pressures, its accuracy may be limited in case of leaks. The minimal predicted increase in lung volume may help to check for accuracy.

## Introduction

In acute lung injury (ALI) and acute respiratory distress syndrome (ARDS), functional residual capacity (FRC) is markedly decreased as a result of numerous factors, including alveolar collapse, pulmonary edema with alveolar flooding, supine position, sedation-induced diaphragm inactivity, and cardiac enlargement [1-5]. Measuring FRC (or end-expiratory lung volume [EELV] when PEEP is applied) might help to measure the aerated lung available for ventilation and to better monitor the effects of ventilation strategies. Reproducible measurement techniques that can be used at the bedside are needed to minimize overdistention and to determine which patients may benefit from recruitment strategies. Repeated CT scans and gas-dilution techniques are two validated methods of lung-volume measurement but are so complex that their use has been confined to research settings. Recently, washout/washin techniques using oxygen [6,7] or nitrogen [8,9] have been made available in ICU ventilators, allowing bedside EELV measurement. A comparison of the nitrogen washout/washin EELV measurement with helium dilution or CT scan had shown good correlations in stable patients ventilated with low-PEEP levels [8]. The limitations of the nitrogen washout/washin technique for EELV measurement under other conditions, such as high FiO_2 _or high PEEP, have not been fully investigated [10].

**Figure 1:**
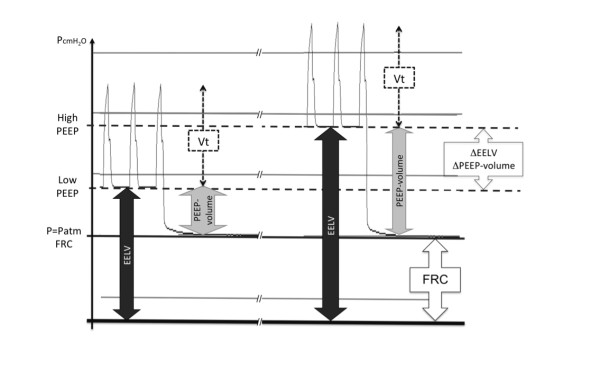
**Schematic representation of the pressure-time diagram at the end of each epoch in a single patient**. Data at both PEEP levels studied are presented. P_atm_, atmospheric pressure; EELV, end-expiratory lung volume measured by using the nitrogen technique; PEEP-volume, volume trapped by PEEP above the functional residual capacity, measured by using a long exhalation to atmospheric pressure (zero end-expiratory pressure); V_t_, tidal volume; ΔEELV, EELV_high PEEP _- EELV_low PEEP _; ΔPEEP-volume, PEEP-volume_high PEEP _- PEEP-volume_low PEEP_.

PEEP-induced changes in lung volume (referred to as PEEP-volume) can also be assessed simply at the bedside by using passive spirometry. This accurate method requires a long expiration to zero end-expiratory pressure (ZEEP), where FRC is assumed to be reached. When considering the changes induced by two different levels of PEEP in a given patient, the difference in EELV (that is, ΔEELV = EELV_high PEEP _- EELV_low PEEP_) should theoretically be similar to the difference in ΔPEEP-volume (PEEP-volume_high PEEP _- PEEP-volume_low PEEP_), assuming that the FRC has not been modified by the PEEP changes (see Figure 1).

We designed a multicenter study with the primary objective of investigating the precision (reproducibility) of the nitrogen washout/washin technique for EELV measurement in patients with ALI/ARDS at two PEEP levels, including a high level, with a small variation in oxygen concentration (10%). Our secondary objective was to evaluate the accuracy of the technique by comparing PEEP-induced changes (Δ) in lung volume with the nitrogen technique or the PEEP-volume above the FRC measured with passive spirometry. As PEEP-volume is relatively easy to measure accurately with a calibrated pneumotachograph, it may therefore be considered a "gold standard." Because we expected possible discrepancies between the two techniques, we also compared the measured changes in lung volume (ΔEELV and ΔPEEP-volume) with the minimal predicted increase in lung volume, computed from static compliance (C_stat_) at low PEEP and ΔPEEP. The minimal predicted increase in lung volume was considered the smallest-volume increase that can occur. We have also used this method to evaluate alveolar recruitment, as described elsewhere [11].

## Material and methods

This was a multicenter study performed in five French medical intensive care units at the Henri Mondor University Hospital in Créteil, European Georges Pompidou University Hospital in Paris, Angers University Hospital in Angers, l'Archet 1 University Hospital in Nice, and Charles Nicolle University Hospital in Rouen. In compliance with French legislation, the institutional review board of the Henri Mondor University Hospital approved the protocol for all centers and waived the need for informed consent, as PEEP optimization was considered part of standard care. The patients or next of kin received information about the study.

### Patients

Patients were enrolled if they met the standard criteria for acute lung injury (ALI) [12]: partial pressure of arterial oxygen over fraction of inspired oxygen (PaO_2_/FiO_2_) less than 300 mm Hg, bilateral pulmonary infiltrates on the chest radiograph, and no clinical evidence of left atrial hypertension. Most patients had ARDS, defined as PaO_2_/FiO_2 _less than 200 mm Hg. Exclusion criteria were age younger than 18 years, pregnancy, history of chronic obstructive pulmonary disease and/or lung surgery, and hemodynamic instability, defined as an increase in vasoactive drug (epinephrine, norepinephrine) dosages in the last 6 hours. All bedside anterior-posterior chest radiographs were reviewed by two independent observers (JJR and QL) according to CT Scan ARDS Study Group criteria to determine the pattern of aeration loss: lobar radiologic hyperattenuation predominating in the lower lobes (focal disease), diffuse radiologic hyperattenuation evenly distributed throughout the upper and lower lobes (white lungs), or patchy radiologic hyperattenuation involving the upper and lower lobes with persistent aeration of part of the upper lobes [13]. Patients with diffuse or patchy aeration loss were classified as having nonfocal disease [14].

### Ventilation strategies

All patients received volume-assist control ventilation by using an Engström ICU ventilator (Version V4 and V5) with a CVOX module sensor (V4.5**) **General Electric, Madison (WI). This ventilator provides bedside EELV measurements by using the multibreath nitrogen-washout technique (MBNW) [8,15-18]. The oxygenation goal was achieved by adjusting FiO_2_, which was maintained constant during the study. Tidal volume was set at 6 ml/kg of predicted body weight. All patients received two PEEP levels, each for 45 minutes, in random order. PEEP levels were set as in the EXPRESS study [19]. In the minimal-distention strategy, PEEP and inspiratory P_plat _were kept as low as possible while keeping arterial oxygen saturation at 88% to 92% or more. External PEEP was set to maintain total PEEP (the sum of external and intrinsic PEEP) between 5 and 9 cm H_2_O. In the optimized recruitment strategy, PEEP was adjusted based on P_plat _and was kept as high as possible without increasing the inspiratory P_plat _above 28 to 30 cm H_2_O. All patients were sedated. Neuromuscular blocking agents were administered only if deemed necessary by the clinician in charge.

### Measurements

#### Lung volume and precision of measurements

At the end of each 45-minute period, blood was drawn for arterial blood gas measurement, and EELV was measured 3 times by using the MBNW technique to assess precision. This technique has been described elsewhere [9,16]. In brief, continuous measurement of end-tidal O_2 _and CO_2 _during a change in FiO_2 _(here, 10%) allows the calculation of nitrogen washout and washin and subsequently of the aerated lung volume. Two assumptions are made: heterogeneity in alveolar gas distribution is considered constant during the measurement procedure, and cellular metabolism and gas exchange between lung capillaries and alveoli are considered stable during the MBNW procedure. The mean of the washout and washin data is computed automatically if the difference between the two is less than 20% (cut-off determined by the manufacturer). Because FRC is a volume measured without PEEP (that is, at atmospheric pressure), we used the term end-expiratory lung volume (EELV) for the volume measured in our study. Three EELV measurements were performed at each PEEP level.

#### PEEP-volume (above FRC) by using passive spirometry

Prolonged exhalation (15 seconds) to the elastic equilibrium volume at ZEEP was performed, at the end of a 45-minute period, to standardize lung-volume history. Pressure and flow were recorded by using a dedicated computer linked to the ventilator (sample every 0.04 seconds), pressure, and flow curves were drawn off-line by using the software (Acknowledge 3.7.3**) Goleta Ca**. Volumes were measured by flow integration. PEEP-volume above FRC was obtained by subtracting the insufflated tidal volume from the flow-signal integration of this long exhalation. PEEP-volume was measured at the end of each of the two PEEP periods.

### Measurement of compliance

C_stat _of the respiratory system was computed by dividing tidal volume by P_plat _(measured during an end-inspiratory pause (1 second)) minus total PEEP. Total PEEP was measured by using an expiratory pause (1 second).

A pressure-volume curve was obtained during low-flow inflation from the low PEEP level to 30 cm H_2_O to check that compliance (C_lin_) was linear or not decreasing within this range.

### Minimal predicted increase in lung volume

The minimal predicted increase in lung volume [20] is the smallest possible lung-volume increase that can be induced by PEEP. It was computed from C_stat _at low PEEP, as follows:

Minimal predicted increase in lung volume (milliliters) *= C*_stat**lowPEEP **_*·*Δ*PEEP*

where ΔPEEP is the difference between high and low PEEP.

This minimal increase should be equal to (if no recruitment occurs) or smaller than (if alveolar recruitment occurs) ΔEELV and ΔPEEP-volume. We evaluated the slope of the pressure-volume curve during tidal inflation to check that compliance did not decrease over tidal inflation and, therefore, that the computed minimal increase was indeed the lowest possible increase that could occur.

### Statistical analysis

All variables are described as median (interquartile range). Precision of the nitrogen technique results was assessed by calculating the coefficient of variation for the three pairs of washout/washin measurements. The coefficient of variation was calculated as the SD of the differences divided by the mean of all measurements. The Bland and Altman method [21] was used to evaluate reproducibility of the nitrogen technique and to evaluate agreement between ΔEELV and ΔPEEP-volume. The largest difference between the three EELV measurements at each PEEP level was plotted against the mean. Accuracy of the technique was assessed by comparing the changes in lung volume induced by the PEEP increase. ΔEELV was plotted against ΔPEEP-volume. Correlations were evaluated by using linear regression (*r^2^*). Paired values were compared by using the Wilcoxon test. The Fisher *t *test and Mann-Whitney *U *test were used when appropriate. Values of *p *smaller than 0.05 were considered significant.

## Results

We studied 37 patients, of whom three were excluded from the analysis because of poor signal quality (two patients had unstable signals during PEEP-volume recording (spontaneous breathing), and one had greater than 20% differences between washout and washin values). Table 1 reports the main characteristics of the 34 patients included in the analysis, 32 with ARDS and two with ALI. Table 2 reports data on ventilation mechanics, ventilator settings, measured volumes, and calculated volumes at each PEEP level. Both PEEP strategies were well tolerated by all patients. No patients experienced any significant desaturation during the study measurements (EELV or PEEP-volume).

**Table 1:** Patient characteristics

	*N *= 34
Age, years	61.0 (45; 72)

Males/Females (*n*)	28/6

SAPS 2	55.5 (35; 65)

Vasoactive agents (*n *of patients/total patients)	20/34

Pulmonary/extrapulmonary cause of ALI/ARDS (number of patients)	26/8

Diffuse/Focal aeration loss (number of patients)	28/6

Ventilation days, median (IQR)	13 (11; 21)

Alive at ICU discharge, number of patients/total patients	22/4

SAPS II, Simplified Acute Physiology Score [32].

**Table 2:** Arterial blood gas values and ventilation during the minimal-distention (low PEEP) and high-recruitment (high PEEP) periods

	Low PEEP	High PEEP	*p *value
pH	7.37 (7.32; 7.44)	7.36 (7.30; 7.41)	0.014

PaO_2_/FiO_2_	135 (106; 175)	174 (122; 220)	< 0.0001

SaO_2 _(%)	95 (93; 97)	97 (95; 99)	0.0001

PaCO_2 _(mm Hg)	41 (36; 46)	42 (36; 48)	0.1

PEEP_tot _(cm H_2_O)	6 (5; 6)	15 (13; 17)	< 0.0001

P_plat _(cm H_2_O)	18 (16; 22)	29 (29; 31)	< 0.0001

C_stat _(ml/cm H_2_O)	33.3 (25.0; 39.9)	28.6 (23.9; 33.8)	0.003

C_lin _(ml/cm H_2_O)	36.0 (26.0; 42.7)	30.0 (24.8; 34.5)	< 0.0001

EELV (ml)	908 (693; 1,180)	1573 (1,025; 1,905)	< 0.0001

PEEP-volume (ml)	186 (120; 261)	815 (473; 1,122)	< 0.0001

C_stat_, static compliance computed as tidal volume/(P_plat _at low PEEP-low PEEP); C_lin_, linear compliance measured on the linear part of the pressure-volume curve; values of *p *for the comparison were calculated for low PEEP versus high PEEP; values are expressed as median (interquartile range).

### Precision of the nitrogen technique

**Figure 2:**
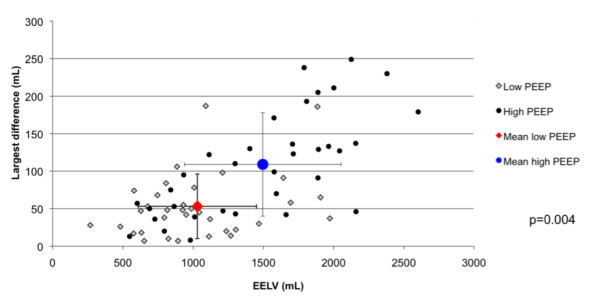
**Largest difference (absolute values) between the three EELV values obtained in each patient, as a function of EELV**. The difference between the measured values was larger at high PEEP (*p *= 0.004). When expressed as a percentage of EELV, no difference was observed according to PEEP level. Gray diamonds, low PEEP; solid circles, high PEEP; red diamond, mean value at low PEEP; blue circle, mean value at high PEEP; vertical and horizontal bars, the standard deviation.

The 34 patients had three pairs of EELV measurements at each PEEP level (that is, 204) (Figure 2). Of these measurements, six (2.9%), in six different patients (two at low PEEP and four at high PEEP) showed greater than 20% differences between washout and washin values and were therefore excluded. The coefficient of variability for the remaining measurements was 3.0% at low PEEP and 3.9% at high PEEP (*p *< 0.0001).

The largest mean difference between the three pairs of EELV measurements was 81 ± 64 ml. The difference was larger at higher PEEP levels (53 ± 43 ml versus 108 ± 69 ml; *p *= 0.004) but was similar when expressed as a percentage of EELV (Figure 2). Mean FiO_2 _was 67 ± 17%; the highest FiO_2 _levels were not associated with greater EELV variability.

### Comparison with PEEP-induced changes in lung volume and accuracy of the method

**Figure 3:**
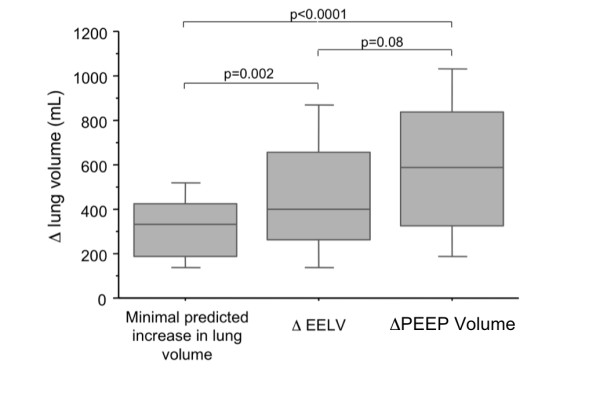
**Median and interquartile range of minimal predicted increase in lung volume, ΔEELV, and ΔPEEP-volume**. Minimal predicted increase in lung volume, 330 (190 to 421) ml. ΔEELV, 402 (263 to 654) ml. ΔPEEP-volume, 585 (325 to 822) ml.

Minimal predicted increase in lung volume, ΔEELV, and ΔPEEP-volume are shown in Figure 3.

**Figure 4:**
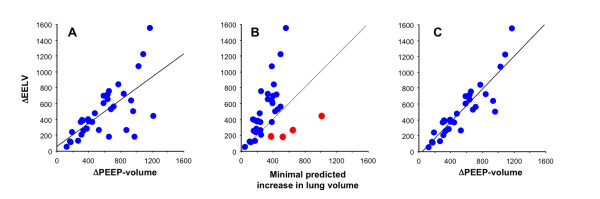
**ΔEELV and ΔPEEP-volume correlation**. **(a) **Correlation between ΔEELV and ΔPEEP-volume in all patients (*r^2 ^*= 0.47). Straight line is correlation: ΔEELV = 62.4 + 0.7 ΔPEEP-volume. **(b) **Relation between the *minimal predicted increase in lung volume *and ΔEELV. Red dots: patients in whom measurement errors were detected; dashed line, identity. **(c) **Correlation between ΔEELV and ΔPEEP-volume after exclusion of the four patients with obvious ΔEELV measurement errors (*r^2 ^*= 0.80). Straight line is correlation: ΔEELV = -42.1 + 1.0 ΔPEEP-volume.

**Figure 5:**
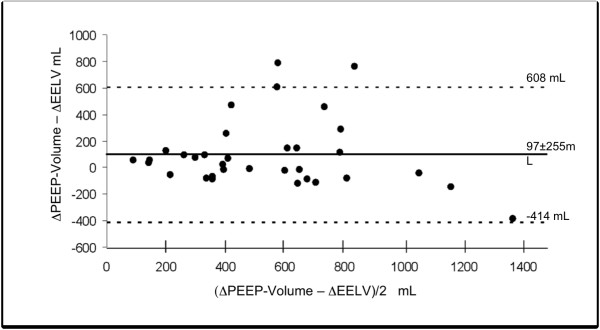
**Comparison according to Bland and Altman **[21]**of measurements of ΔPEEP-volume and ΔEELV**. Bias between the two methods was 97 ± 255 ml with a 95% confidence interval for the limits of agreement (dashed lines) of -414 to 608 ml.

ΔEELV and ΔPEEP-volume were only modestly correlated with each other (Figure 4a) (ΔEELV = 62.4 + [0.73 ΔPEEP-volume]; *r^2 ^*= 0.47). Bias between these two measuring methods was 97 ± 255 ml, with a 95% confidence interval for limits of agreement of -414 to 608 ml (Figure 5).

The relation between the minimal predicted increase in lung volume and ΔEELV was dispersed (Figure 4b). In particular, four patients had ΔEELV values that were substantially lower than the minimal predicted increase in lung volume (red dots; Figure 4b), suggesting underestimation of the volume change by EELV measurement. All four patients received PEEP levels ≥ 16 cm H_2_O, compared with only five of the 30 remaining patients (*p *= 0.003), and three had focal aeration loss compared with only three (10%) of the 30 remaining patients (*p *= 0.01). FiO_2 _was high (80% ± 16%) in these patients but was not significantly higher than that in the other patients (*p *= 0.1). The cause of ARDS (pulmonary or extrapulmonary) was not associated with measurement discrepancies. The high PEEP values suggested possible occurrence of leaks that could invalidate the measurements. When we excluded these four patients whose ΔEELV values were lower than the predicted minimal increase in lung volume, the correlation between ΔEELV and ΔPEEP-volume became substantially stronger (*r^2 ^*= 0.80; Figure 4c).

## Discussion

The main results of this physiological study can be summarized as follows: (a) the MBNW technique at two PEEP levels provided reproducible EELV measurements with acceptable precision; and (b) compared with ΔPEEP-volume and the minimal predicted increase in lung volume due to PEEP, ΔEELV measured by using the nitrogen technique seemed accurate for measuring lung-volume variations induced by PEEP. In a few patients, however, the method could give erroneous results, especially in case of high pressures. Comparing with the minimal predicted increase in lung volume may help to detect these errors.

### Nitrogen technique variability

The MBNW technique described by Olegard *et al*. [9] allows bedside EELV measurement by using small and safe FiO_2 _increases and decreases (± 10%). Precision was greater with larger FiO_2 _changes [8,16], because nitrogen changes were greater. The small (10%) FiO_2 _change used in our study may have contributed to the test-retest variability but was deemed safer for our hypoxemic patients. All measurements were performed at the steady state 45 minutes after a change in PEEP, and no other interventions likely to affect cardiac output were performed, the patients being considered stable. Fewer than 3% of the EELV measurements failed (greater than 20% difference between washout and washin). Because the technique used to measure EELV involves computing the mean of washin and washout values [9], we assessed test-retest variability without comparing washout with washin. The variability we found in patients with ALI or ARDS at each PEEP level was comparable to that reported by Olegard *et al*. [9], who studied chiefly postoperative patients. As with the helium-dilution technique, absolute variability of the nitrogen technique in our study increased with higher PEEP and higher EELV. However, variability relative to absolute lung volume did not differ for higher EELV values (Figure 2). The lower precision reported by the manufacturer for FiO_2 _> 70% was not replicated here, but the flawed measurements seemed to occur at higher FiO_2 _values.

### PEEP-induced changes in lung volume

EELV values at low PEEP in our study were very low (less than 1,000 ml at low PEEP) and similar to values obtained previously by using CT scan [2,22] or helium dilution [23] in ARDS patients. PEEP-volume and EELV represent different volumes obtained with two totally independent methods. We thus compared lung-volume changes induced by PEEP. ΔEELV and ΔPEEP-volume; both evaluated the PEEP-induced lung volume increase. The correlation was good in some patients but poor in others (Figure 5). The variability of EELV values may have contributed to a poor correlation. We sought to detect obviously flawed data by using a third method. Katz *et al*. [20] demonstrated that the lung-volume increase induced by PEEP changes was larger than expected from the airway-pressure change and compliance at low PEEP, indicating progressive lung recruitment [11]. We therefore calculated the minimal predicted increase in lung volume induced by PEEP, which is easily derived from C_stat _at low PEEP [20]. In addition, by tracing a pressure-volume curve over the tidal-volume range at low PEEP, we checked that compliance did not decrease significantly within this volume range, to ensure that no volume increase smaller than the calculated minimal increase could occur. This method might prove useful at the bedside to assess the lower ΔEELV limit. Any difference between ΔEELV and this minimal predicted increase in lung volume may be considered an estimate of alveolar recruitment [11]. ΔPEEP-volume may slightly underestimate the lung-volume change, because of the assumption that FRC is unchanged after exhalation from high or low PEEP (Figure 3). Yet recent data [24] suggest that FRC may increase after high PEEP compared with low-PEEP ventilation. We used a 15-second expiration to ZEEP to minimize this problem. Our analysis, made at two PEEP levels, shown elsewhere, suggested that FRC was stable for our measurements [11].

Obvious discrepancies occurred in four patients. All four patients had the highest set PEEP levels (> 16 cm H_2_O). Although not proven, it is very possible that microleaks due to high set PEEP may explain discrepancies by decreasing the EELV*_high PEEP _*measurement and therefore ΔEELV. The higher set FiO_2 _values in these four patients may have adversely affected measurement precision, although further studies are needed to evaluate this possibility. Patients with focal aeration loss are at higher risk of hyperinflation versus recruitment [25], and the lung-volume distribution due to PEEP depends closely on disparities in regional lung compliance [26]. Another hypothesis could be that EELV discrepancies in patients with higher PEEP and focal aeration loss may be related to differences in regional gas distribution. MBNW equilibration may be impaired by regional time-constant inequalities [27], and a higher dead space due to higher PEEP [28] and hyperinflation [29-31]. In clinical practice, we suggest comparing the increase in EELV with PEEP to the minimal predicted increase in lung volume to detect erroneous measurements.

## Conclusions

The MBNW technique exhibits acceptable accuracy and precision for lung-volume measurement at different PEEP levels in patients with ARDS. Substantial underestimation of lung-volume changes may occur, at least in some patients, presumably in case of leaks due to high pressures, and additional measurements may be required to check this accuracy.

## Key messages

• Nitrogen washin/washout technique exhibits acceptable accuracy and precision for lung-volume measurement at different PEEP levels and high FiO_2 _in patients with ARDS.

• Underestimation of lung-volume changes may occur in some patients presumably in case of leaks due to high pressures.

## Abbreviations

ALI: acute lung injury: ARDS: acute respiratory distress syndrome; C_lin_: linear compliance; C_stat_: static compliance; EELV: end-expiratory lung volume; FRC: functional residual capacity; MBNW: multibreath nitrogen washout; PEEP: positive end-expiratory pressure; PEEP-volume: trapped lung volume due to PEEP; P_plat_: plateau pressure; V_t_: tidal volume.

## Competing interests

JD, LB, JCMR, AM, and their institution are involved in a patent with General Electric describing a method used to estimate alveolar recruitment. A grant was also received from General Electric for the conduct of the study. General Electric had no access to the data or to the content of the manuscript. All authors kept full control of the analysis of the data and the writing of the manuscript. JM and his team are currently doing research regarding FRC measurements, which is sponsored by a GE grant. NL, CS, GB, JLD, FDM, JJR, QL, and GB declare that they have no competing interests.

## Authors' contributions

JD designed the study, and participated in data acquisition, statistical analysis, interpretation, and wrote the manuscript. NL participated in study design, data acquisition, statistical analysis, and manuscript editing. CS participated in data acquisition. GB participated in data acquisition. FDM participated in study design. AM, JCMR, JLD, and GB participated in study design and manuscript editing. JM participated in manuscript editing. JJR and QL participated in data analysis and manuscript editing. LB participated in study design, data analysis and interpretation, and manuscript writing. All authors read and approved the final manuscript.
